# Virtual Fence Responses Are Socially Facilitated in Beef Cattle

**DOI:** 10.3389/fvets.2020.543158

**Published:** 2020-09-30

**Authors:** Hamideh Keshavarzi, Caroline Lee, Jim M. Lea, Dana L. M. Campbell

**Affiliations:** Agriculture and Food, Commonwealth Scientific and Industrial Research Organization (CSIRO), Armidale, NSW, Australia

**Keywords:** facilitation, group-living, GPS, behavior, allelomimicry

## Abstract

Group-living can be socially advantageous where the behavior of individuals may be modified by group members through socially facilitative processes. Virtual fencing contains cattle by providing audio and electrical signals via a neckband device. However, little is known about social influences on learning to appropriately respond to the virtual fence (VF) cues. This study aimed to determine whether cattle respond to the behavior of conspecifics during their initial interactions with a VF across 3 days. Sixty-four Angus steers, naïve to virtual fencing, were placed into 8 paddocks (8 animals/group), divided with a VF into two areas- an inclusion and exclusion zone. The animals received an audio cue if they approached the VF followed by an electrical pulse if they continued into the exclusion zone. The GPS and audio and electrical stimuli data were recorded. To quantify social facilitation, individual VF interactions were grouped into 179 “events” across 3 days; starting from when the first animal (leader) approached the VF. The responses of other animals were categorized as (1) followed the leader to move into the exclusion zone (followers, F), (2) accompanied the leader back into the inclusion zone (facilitated, Fa), (3) did not show any reaction (non-facilitated, NFa). A social facilitation score (SFaS) was calculated as SFaS (%) = (F/(Fa+NFa+F)) ^*^ 100. A single leader animal led on average 37% of events with 76.2% of all reactions categorized as facilitated by other individuals. Animals responded to the behavior of conspecifics more during the VF implementation compared with facilitated movement during natural grazing patterns when no VF was present (*P* < 0.001). On average, cattle stopped or turned away to 3.8 (± 2.9 SE) audio cues before ever receiving their first electrical pulse. There was a positive correlation (*R* = 0.34, *P* = 0.006) between the number of audio cues received prior to the first electrical pulse and the proportion of all audio cues that were not followed by an electrical pulse. In conclusion, cattle stayed within the inclusion zone based on the response of conspecifics, including some social impacts on individual rates of associative learning between the audio and electrical cues.

## Introduction

Social animal species live in groups which is thought to have several advantages for predator protection, improved foraging success (1) and may confer other social benefits such as keeping warm, mate access (2), allo-grooming (3, 4), and improved reproduction through maternal kinship (5). Although some individuals may move away from the group or vary in their proximity to other individuals (6) in group-living animals, there are collective processes occurring and the individuals operate under consensus decisions (7). That is, while all animals are acting autonomously, they typically follow one or a few leaders resulting in coordinated group movements (8, 9). The influence of animals on moving group members into new areas can be related to their dominance status, age, or position in a social network (10, 11). Animal species in groups can also be influenced by conspecifics through watching or interacting with other individuals which can facilitate choosing what food to eat, or specifically how to access it, and predator avoidance (12, 13). There are multiple types of defined processes regarding the social transmission of behavior and information with varying degrees of evidence across different livestock species [reviewed in (14)]. The process of social facilitation (also called “allelomimicry” or “contagious behavior”) is a term commonly used to define a situation where the behavior of one individual instigates the same behavior in another individual (14). Social facilitation is in contrast with social learning where an individual is stated to have socially learned a new behavior if it is retained when the demonstrator is absent (14).

Cattle typically live in groups of differing sizes, both in rangeland environments and more intensive farm herds. Within these groups there is evidence for social relationships between individuals (15, 16), differences in dominance status (6, 10), leaders and followers during grazing movements (11), and effects of social rank on milking patterns in automatic milking systems (17, 18) and positions at feed troughs (19). Cattle will demonstrate social facilitation (or allelomimicry) of postural behaviors such as greater synchronization of lying between neighboring individuals within a group (20) and synchronization of time budgets of different cattle breeds at pasture (21). Cattle will also show synchronized drinking behavior (22) and will graze specific toxic weeds if placed in paddocks with other cattle that readily consume them, including modifying previous correct aversions to the toxic plant (23, 24). The influence of social facilitation could thus be extended to other contexts of cattle farming such as the acclimation to and learning of new technologies.

In modern farming practices, new automated technologies such as automatic milking systems have changed livestock management (25). Livestock are expected to learn and respond appropriately to new farming environments and technologies (26) but learning may not be equal between all individuals resulting in culling of animals that do not adapt (27). Automated virtual fencing (VF) is a new agricultural technology that may transform the grazing livestock industry. Animals are restricted in a specified area via receiving stimulatory cues rather than through the presence of a physical fence (28) enabling remote animal monitoring and movement control. In the eShepherd® system (Agersens, Melbourne, VIC) all cattle wear a neckband device that will administer an audio tone as the animal approaches the VF, and an electrical stimulus if the animal continues moving forward. Cattle exposed to a VF show two stages of learning to avoid receiving electrical stimuli. Firstly, the cattle show avoidance learning where they rapidly learn to stay within the specified inclusion zone rather than continuing to move farther into the exclusion zone where they receive repeated audio/pulse combinations. This is followed by associative learning where they learn to respond appropriately to the audio cue alone (29, 30). However, individual cattle within the groups vary greatly in their rates of both avoidance and associative learning (29, 30) which could impact their adaptation to the technology (31). This individual variation may in part be a result of social influence. Campbell et al. (29, 30) found that cattle exposed to a VF for the first time learned to stay within the inclusion zone and respond to the audio cue alone, however, some cattle turned away from the audio cue without having first experienced the electrical stimulus suggesting social facilitation was occurring. It is currently unclear how social factors may affect cattle responses to a VF system. If cattle interact with the VF as a group during the initial stages of exposure, then social facilitation may improve the responses of some individuals resulting in 100% herd adaptation to the technology where all animals correctly remain in the inclusion zone. Alternatively, social facilitation may result in only some animals (leaders) being required to wear the neckband devices.

The current study aimed to look at the pattern of social facilitation during the first 3 days of VF activation by (1) identifying individuals that first approached the fence (leaders) within the groups, and (2) quantifying the degree of social facilitation when avoiding the VF boundary.

## Materials and Methods

### Ethical Statement

The experiment was approved by the CSIRO FD McMaster Laboratory Chiswick Animal Ethics Committee (ARA18/25).

#### Experimental Design

**Figure 1:**
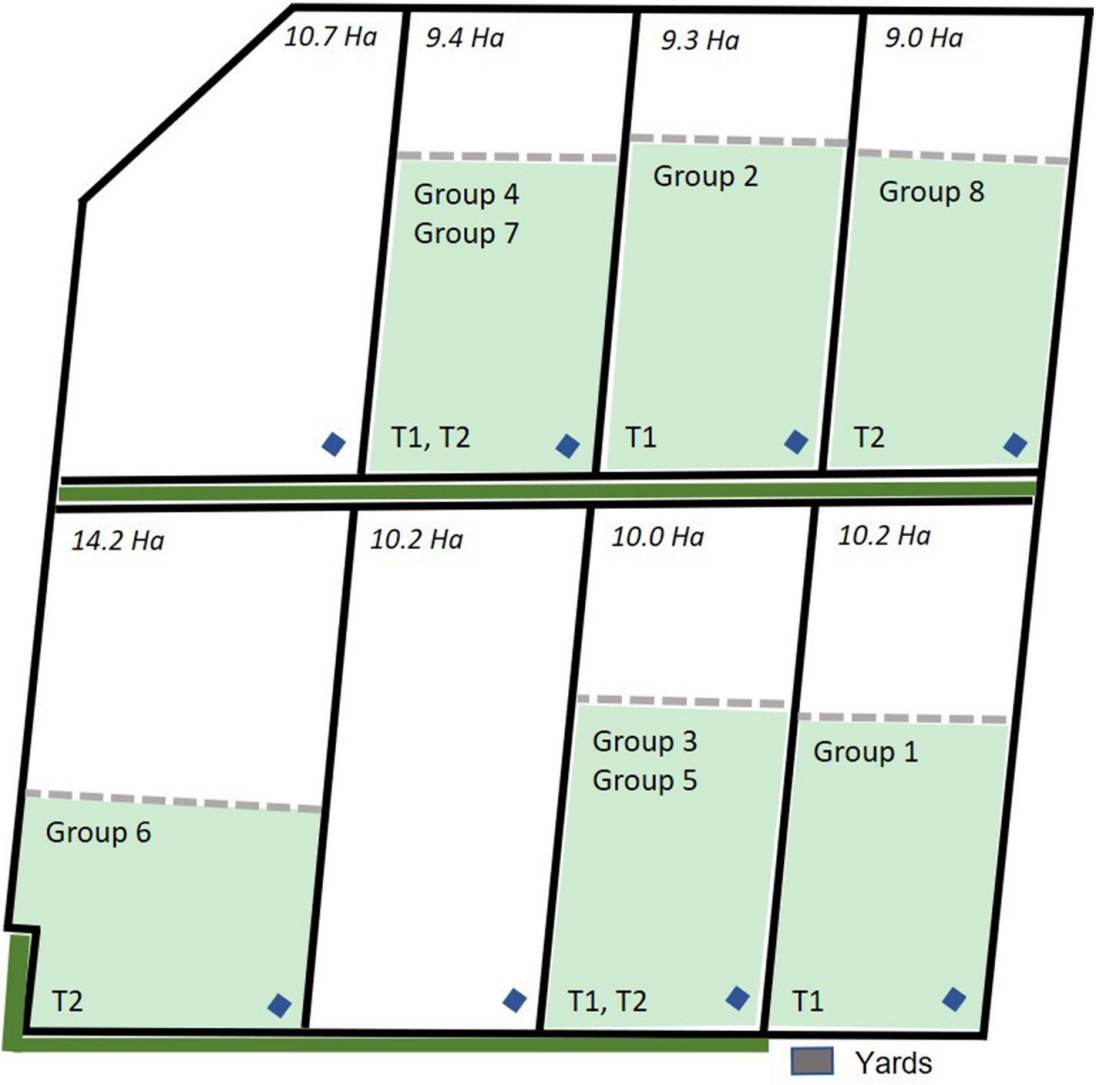
Map of the group allocation to the paddocks across the trial period for groups 1 to 4 that were included in the first trial (T1) and groups 5 to 8 that were included in the second trial (T2). Paddock size (ha), location of the yards, water points (blue diamonds), tree line (solid green line) outside the paddock physical fences, and the placement of the virtual fence (dashed line) is indicated. Each fenced inclusion zone was 6 ha in size. Map is adapted from Campbell et al. (29).

For this study, the data collected across 3 days each from 8 groups of eight 12 to 14-month old Angus steers (n = 64 animals) with an average starting body weight of 405 ± 31.8 kg were used. All cattle were naïve to virtual fencing for the data collection period of the current study. Data were collected from the 8 groups in a staggered method across a period of 3 months as there were limited numbers of both neckband devices and available paddocks to test all groups simultaneously. Additionally, groups 1–4 were part of a larger trial assessing the behavior and welfare of cattle exposed to electric tape or virtual fences conducted at the CSIRO Chiswick site in Armidale, NSW from January through March 2019 and full details of that experimental protocol can be found in Campbell et al. (29). This study used data from the first 3 days of VF exposure during the larger trial for groups 1 to 4 and is referred to as the “first trial.” Groups 5–8 were those cattle that were exposed to electric tape during the larger trial but were then exposed to a VF for the first time 3 days immediately following the conclusion of the larger trial and these groups are referred to as the “second trial.” Briefly, all cattle were fitted with eShepherd® neckbands that carried the virtual fencing device. Animals were placed into separate paddocks 9–14 ha in size with groups 1 and 2 placed in January 2019, groups 3 and 4 in February 2019, and groups 5–8 in March 2019. Paddocks were grassed with a tree line at one edge of 7 of the 8 paddocks as indicated in Figure 1. The average (mean ± SD) temperature (T) and relative humidity (RH) across the trial period (3 days) were: T = 21.43 ± 0.41°C, RH = 72.76 ± 2.92% for groups 1 and 2; T = 16.50 ± 0.65°C, RH = 72.43 ± 2.45% for groups 3 and 4; T = 16.73 ± 3.21°C, RH = 76.83 ± 9.71% for groups 4 to 8 based on weather data collected directly at the Chiswick site.

All groups had an adaptation period to the paddocks with free access to the entire paddock area for 9 days. However, the groups in the second trial (groups 5–8) were placed back into the test paddocks and a virtual fence line was set the following morning to commence data collection. This timeline was selected as two of the groups had just spent the past 5 weeks in the paddock and the other two groups had previously spent 5 weeks in the paddocks as the electric-tape exposed groups from the larger trial presented in Campbell et al. (29). Following adaptation, single, straight, virtual fence boundaries were specified using GPS coordinates, and each paddock was divided into two areas—inclusion and exclusion zones. The inclusion zone was ~6 ha in size across all paddocks (Figure 1). GPS coordinates of the virtual fence were transmitted to the unit using a radio frequency link. The animals received the audio cue if they approached the virtual fence. After receiving the audio cue, the animals could respond to the audio cue and stop and/or turn back to the inclusion area, or continue moving forward, in which case they received a short sharp pulse through the unit [further descriptions of the virtual fencing algorithm are reported in (29, 30, 32, 33)]. This sequence of an audio cue followed by the electrical pulse was repeated if the animal walked through the fence line and continued into the exclusion zone, but all cues ceased when an animal turned around to walk back out of the exclusion zone. The device had a safety limit for the number of consecutive pulses an animal received if it was continuing to move farther into the exclusion zone or it was moving above a specified velocity (i.e., running) but precise details on these functions are commercial in confidence. The device also included a “grazing function” to account for animals that may gradually encroach upon the VF by grazing. The natural behavioral pattern of grazing can mimic the correct response by the animal to the neckband cues where they may stop after receiving an audio cue during their slow grazing movement forward. Therefore, if an animal gradually moved into the exclusion zone and was not turning around when it received the audio signal, after 3 consecutive audio cues an electric pulse was applied. Each group of 8 animals was exposed to the virtual fence for 3 days which was the sampling period for this study [this was the minimum exposure time for groups 1–4 as their responses to a VF were recorded for 4 weeks as part of (29)].

#### Data

The time-stamped GPS positional data which recorded approximately every second when the animal was near the fence line and/or walking/grazing were downloaded from the neckband device. All audio cue and electrical pulse data of individual animals across 3 days of fence activation were also downloaded from the neckband device. One day of GPS data from the last day of the habituation period for each group was also included for control comparisons of social facilitation of movement patterns when no VF was present. Data editing was carried out in the SQL server (34). To prepare the dataset for analysis of social facilitation via GPS movement patterns and cues received, the original VF dataset was edited to eliminate records of: (1) before the first animal moved into the exclusion area, and (2) data during the night as it was assumed visual contact during learning would be limited. Thus, across 3 study days, the data used were based on sunrise and sunset in Armidale at the time of investigation for each group as follows: from 6 a.m. to 8 p.m. (groups 1 and 2), from 6:45 a.m. to 7:30 p.m. (groups 3 and 4), and from 7 a.m. to 7 p.m. (groups 5–8). For the first day, the time of the first interaction with the VF in each group (varied from 8 a.m. to 3:30 p.m. across the groups) was considered the starting time point. The control dataset for each group was of the same time periods across 1 day prior to activation of the VF.

#### Social Influences Analyses

##### Control observations

To compare the degree of social facilitation of movement patterns in the absence of a VF the movement of each group was scanned every 30 min across 1 day (daylight period only) until an instance was identified where the animal at the front of the group (termed the “leader”) turned back in a different direction. The movements of the other individuals were then observed for up to 5 min to identify reactors (R—those animals who followed the leader in the same direction) and non-reactors (NR—those animals who did not follow the leader's direction change). A period of up to 5 min was selected as this was the maximum duration of the majority of VF events (Table 1). A total of 12 instances were identified for each group across the day which resulted in a power analysis equal to 0.99 in total across all groups (*n* = 96) and 0.7 within groups (*n* = 12). This following behavior was quantified into a percentage “social following score” for each event using the below equation:

$$\begin{array}{l} Social\;Following\;Score\;\left(SFoS,\;\%\right)=\\ \;\left(\frac{Reactors}{Reactors+Non-reactors}\right)*100 \end{array}$$

**Table 1:** The percentage summary of VF interaction events of different durations (min) for 8 cattle groups^a^.

		**Duration of events**		
**Study groups**	**Total events (*n*)**	**1–5 min (%)**	**6–10 min (%)**	**11–15 min (%)**
Group 1	36	58.3	36.1	5.6
Group 2	16	56.3	37.5	6.2
Group 3	7	71.4	14.3	14.3
Group 4	27	44.4	40.8	14.8
Group 5	19	68.4	26.3	5.3
Group 6	16	50.0	25.0	25.0
Group 7	30	50.0	33.3	16.7
Group 8	28	42.9	42.8	14.3
Total/Mean	179	55.2	32.0	12.8

a *Total events for each group was calculated based on interactions with the VF across 3 study days*.

##### Leadership during VF events

In this study, the term “leadership” is used to define the first animal(s) who interacted with the VF and received signals for each separate interaction event. To quantify leadership during fence interactions, the group movement behavior was plotted in R (35) using the “ggplot2 package” (36) for each of the 8 groups during their first experience with the VF to describe initial group reactions to the stimuli. Leadership for each subsequent interaction was then determined across separate VF events. An event started from first contact with the VF where an animal received an audio cue only or an audio cue followed by an electric pulse. The event duration was then defined as from the time when the first animal touched the fence and at least one other animal reacted to their interaction until either (1) all animals moved away in distance from the virtual fence and had no more interactions (2) a minimum of 10 min had elapsed between when the last animal interacted and the first animal interacted in a new event, or (3) all animals had turned away from the direction of the virtual fence and then turned back toward it. Each event lasted up to 15 min; only 12.8% of all events were between 10 and 15 min duration (Table 1) with 3 events reaching 15 min where either the cattle broke through the fence and ventured far into the exclusion zone, or cattle were continuing to interact with the fence and receive signals during the first day of exposure. Typically, the cattle were grouped together and thus more than one individual interacted with the fence sequentially. There were only a few instances (*n* = 5) where an isolated individual touched the fence and received a signal and no other animals were near it and these data were excluded from the analyses. Across the 3 days for the 8 groups, a total of 179 separate events were recorded and the leader animal (s) identified (Table 1).

##### Social facilitation

For analysis of social facilitation, first the behavior of other individuals relative to the leader (s) during each VF interaction event (excluding the first event) was quantified. After the leader's interaction, the other animals within the group might (1) follow the leader into the exclusion zone, (2) follow the leader back into the inclusion zone, or (3) act independently of all the leader's movements. The animals' reactions (defined as movement in a backward or forward direction relative to the VF) were monitored for a time period up to 15 min (Table 1 displays the durations of identified events). In total, 171 events (of a total 179 events as the first event for each group was excluded) for animals in all groups across 3 study days were plotted in the R “ggplot2 package” (36) to look at the individual movement direction and the group's behavior to quantify how many animals moved back into the inclusion zone as a result of receiving a cue themselves or from watching the others (socially-facilitated). The animals were considered to have been socially-facilitated if they moved back into the inclusion zone without receiving a signal themselves within that particular event and when at least some of them had had previous experience with the VF. Data to study the social facilitation of the VF, therefore, was limited to the second event onwards when at least some animals had experienced the VF. The responses of other animals in the group were assessed in terms of their movement pattern (heading forward or turning back) within the group relative to the VF and position relative to other animals to indicate if they were staying within the inclusion zone based on the leader animals' interaction with the VF. In summary for each event the animals were considered as below:

Leader (s): The animal (s) who touched the VF first and received the signal as an audio cue or audio cue/electric pulse combination for each event.
Follower (s): The animal (s) who followed the leader to touch the VF/move into the exclusion zone with a time interval of at least 1 min after the leader(s) touched the VF.
Facilitated: The animal (s) who moved back into the inclusion zone as a result of accompanying the leader and his followers moving back into the inclusion zone without receiving any signal themselves.
Non-facilitated: The animal (s) who were close to the leader and his followers but did not change their movement direction to accompany them back into the inclusion zone nor did they interact with the VF.

Social facilitation score (SFaS): This is defined in this study as the proportion of animals who moved back into the inclusion zone as a result of the behavior of others without receiving a signal for each event. This was calculated as per below:

$$\begin{array}{l} Social\;Facilitation\;Score\;\left(SFaS\;\%\right)=\\ \;\left(\frac{Facilitated}{\left(Facilitated+Non-Facilitated+Followers\right)}\right)*100 \end{array}$$

The animal(s) who were clearly separated from the rest of the group (based on visual inspection of the GPS plots) or whose movement path was in the opposite direction of the leader and VF line (mean of 88 m away from the main cluster; range: 40–240 m) at the time of an event were not considered in the social facilitation score calculation. In addition, all animals in groups 3, 4, and 7 had experience with the VF during the first interaction i.e., all “facilitated animals” were 100% experienced from the second interaction onwards. While the range of experience with the VF for facilitated animals in the second interaction for other groups were: group 1 = 62.5%, group 2 = 28.5% (one animal in group 2 was far away from the others on the first day and was not considered in this calculation), group 5 = 25%, group 6 = 75%, and group 8 = 87.5%.

The SFaS during the VF events for each study group for those events that were up to 5 min duration was compared with the SFoS from the identified control events using a unpaired two-tailed *t*-test (due to unequal events numbers for the control and test periods for each group) with α set at 0.05. For the overall comparison of SFaS and SFoS in which the average of these two parameters for study groups was used, the comparison was performed using a paired *t*-test. The percentage values were arcsine-transformed to meet the assumption of normality, but the raw values are presented in the results. In addition to quantifying the social facilitation during avoidance of the VF, the number of audio cues each animal received prior to receiving their first electric pulse were calculated to determine how social facilitation affected the associative learning between the audio cue and electrical pulse. These were calculated across the full dataset (including nighttime hours).

Finally, a Spearman correlation between the number of received audio cues before the first pulse and the proportion of “audio-only” cues (i.e., the proportion of all received audio cues that were not followed by a pulse) across 3 study days (nighttime also included) across each individual animal was estimated using the “ggpubr” package in R (37).

## Results

### Leadership During VF Events

**Figure 2:**
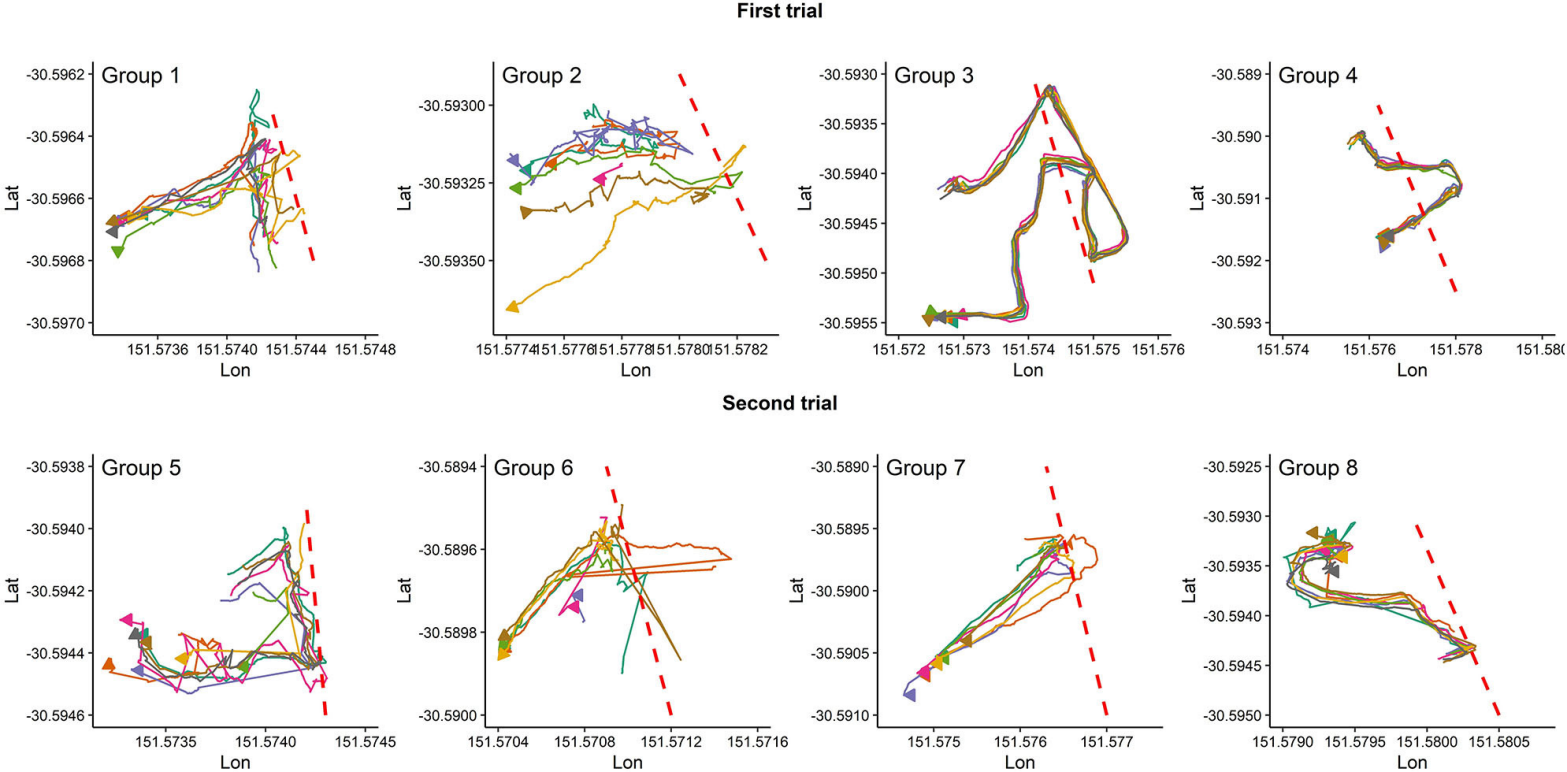
Individual animal movement behavior during the first time interacting with the VF fence (red dashed line) for each group of 8 cattle. Plots are drawn across a period of 10 min, individual cattle are represented by separate colors, and the direction of the arrow indicates direction of travel at the conclusion of the 10 min period.

Figure 2 presents the pattern of moving into the exclusion zone for the first time after the VF was activated for all studied groups. Overall, animals in each group behaved differently in whether they followed the leader animal (s) to move farther into the exclusion zone or back into the inclusion zone. For instance, all animals in groups 3, 4, and 7 received signals during the first interaction with the VF but animals in group 7 responded to the received signals by turning back into the inclusion zone while those in groups 3 and 4 broke the fence and moved farther into the exclusion zone, returning to the inclusion zone 10 min later. For the rest of the groups, the percentage of animals who received signals during the first interaction with the VF varied from 12.5% in group 2 (only the leader touched the fence at the first interaction) to 62.5% in group 5 (Figure 2).

Table 2 presents the percentage contributions of leader animals across 3 study days in the 8 investigated groups. The information is presented in terms of total events that occurred and the percentage of events led by leader animals (one, two, or three) over the study period. Overall, one leader animal (alone or as a part of up to 3 leaders) in all groups led on average, 37% (varied from 27.8 to 71.4%) of events. Increasing the number of leader animals to two and three leaders covered 59.5% (varied from 42.8 to 100%), and 74.8% (varied from 60.7 to 100%) of events, respectively. The variance between groups in number of VF interactions (varied from 7 to 36 in the first trial vs. 16 to 30 in the second trial), and leadership contribution (e.g., varied from 31.2 to 71.4% in the first trial with one leader vs. 31.2 to 43.3% in the second trial with one leader) was less in the second trial compared to the first one (Table 2).

**Table 2:** The percentage of virtual fence interaction events led by specific animals across 3 study days for 8 cattle groups.

		**Events led by individual animals (%)^b^**		
**Groups^a^**	**Total events**	**One leader**	**Two leaders**	**Three leaders**
**First trial**				
Group 1	36	27.8 (steer 3)	52.8 (steer 3, 1)	69.4 (steer 3, 1, 14)
Group 2	16	31.2 (steer 12)	50.0 (steer 12, 11)	68.7 (steer 12, 11, 13)
Group 3	7	71.4 (steer 24)	100.0 (steer 24, 23)	100.0 (steer 24, 23)^c^
Group 4	27	29.6 (steer 28)	48.1 (steer 28, 19)	69.9 (steer 28, 19, 31)
**Second trial**				
Group 5	19	36.8 (steer 32)	63.1 (steer 32, 37)	84.2 (steer 32, 37, 13)
Group 6	16	31.2 (steer 2)	56.2 (steer 2, 19)	68.7 (steer 2, 19, 36)
Group 7	30	43.3 (steer 26)	63.3 (steer 26, 3)	76.7 (steer 26, 3, 12)
Group 8	28	25.0 (steer 24)	42.8 (steer 24, 25)	60.7 (steer 24, 25, 9)
Mean	22.4	37.0	59.5	74.8

a *Groups 1–4 belonged to the first trial (1, 2: cohort 1, and 3, 4: cohort 2) and groups 5–8 belonged to the second trial (single cohort)*.

b *The leader animals were the individuals who touched the fence first for each particular event across 3 study days*.

c *Group 3 already reached 100% of events with only two leader animals*.

### Social Facilitation

A total of 171 events (without considering the first interaction with the VF for each group) across 3 days for 8 groups were identified. On average, 76.2% of animals avoided the VF based on the behavior of other individuals which varied from 72.8% in group 8 to 80.5% in group 3 (Table 3). The percentage of social facilitation (SFaS) and number of interaction events fluctuated across the groups. Except for animals in group 4 and group 6, VF interactions had decreased by day 3. Overall, animals in group 1 and group 3 had the most and fewest mean VF interactions, respectively (group 1 mean = 11, group 3 mean = 2; Table 3). In terms of social facilitation, the percentage of animals who avoided the VF based on other individuals' interactions increased at the end of the study in over half of the groups, but decreased for groups 2, 3, and 5 resulting in all groups having similar mean social facilitation percentages across the 3 study days. Overall, the SFoS during control events (mean = 52.6%) was significantly (*df* = 7, *t* = −9.57, *P* < 0.001) lower than the SFaS (mean = 80.5% for events with up to 5 min duration) during VF events but variation in the SFoS/SFaS difference was present across the 8 groups (Table 3).

**Table 3:** The summary of VF interaction events and social facilitation percentage of animals across 3 study days for 8 cattle groups including control comparisons.

	**Study groups**							
**Items/day**	**1**	**2**	**3**	**4**	**5**	**6**	**7**	**8**
**Event no**.								
Day 1	9	7	3	5	10	5	8	12
Day 2	20	5	2	13	3	1	15	10
Day 3	6	3	1	8	5	9	6	5
Total	35	15	6	26	18	15	29	27
SFoS^a^	54.7	57.1	54.4	44.0	56.3	50.0	60.7	61.2
**SFaS (%)^b^**								
Day 1	69.6	91.2	100.0	73.4	73.0	59.0	75.6	70.9
Day 2	77.4	57.0	70.0	68.1	84.1	100.0	71.7	72.4
Day 3	88.7	79.4	71.4	82.9	69.8	70.0	78.6	75.2
Mean^c^	78.6	75.9	80.5	74.8	75.6	76.3	75.3	72.8
df^d^	29	19	15	22	23	18	17	22
*t*-value	−2.61	−1.36	−2.59	−5.28	−3.11	−1.61	−1.35	−0.48
*P-*value	0.01	0.18	0.02	<0.01	0.004	0.12	0.19	0.63

a The social following score (SFoS) was calculated from control events as SFoS (%) = (R/(R+NR)) *100 where R (reactors) = animals who followed the leader in the same movement direction, and NR (non-reactors) = animals who did not follow the leader.

b The social facilitation score (SFaS) was calculated from VF events as SFaS (%) = (Fa/(Fa+NFa+F)) *100 where Fa (facilitated) = animals who accompanied the leader back into the inclusion zone, NFa (non-facilitated) = animals who did not show any reaction in terms of following the leader to move into the exclusion zone or accompanying him back into the inclusion zone, and F (followers) = animals who followed the leader into the exclusion zone.

c *SFoS from control events up to 5 min duration was compared with the SFaS across all 3 study days with VF events that were up to 5 min*.

d *Degrees of freedom*.

*The first event was not considered in this table*.

**Figure 3:**
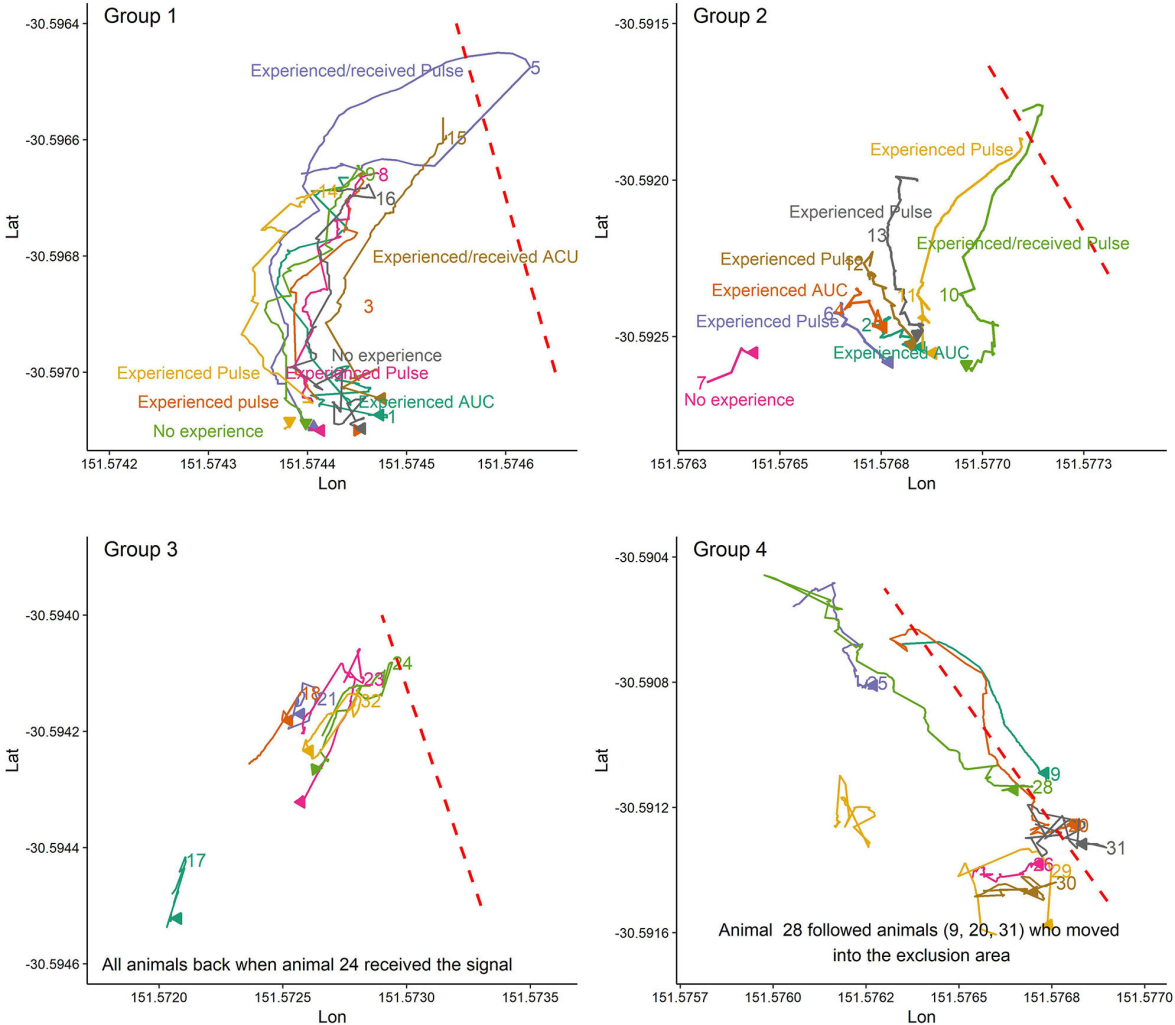
The pattern of avoidance of the VF and exclusion zone as facilitated by others who reacted to the received cues and moved back into the inclusion zone when some animals had, and some animals did not have experience with the VF. Plots are drawn across a period of 5 min.

**Figure 4:**
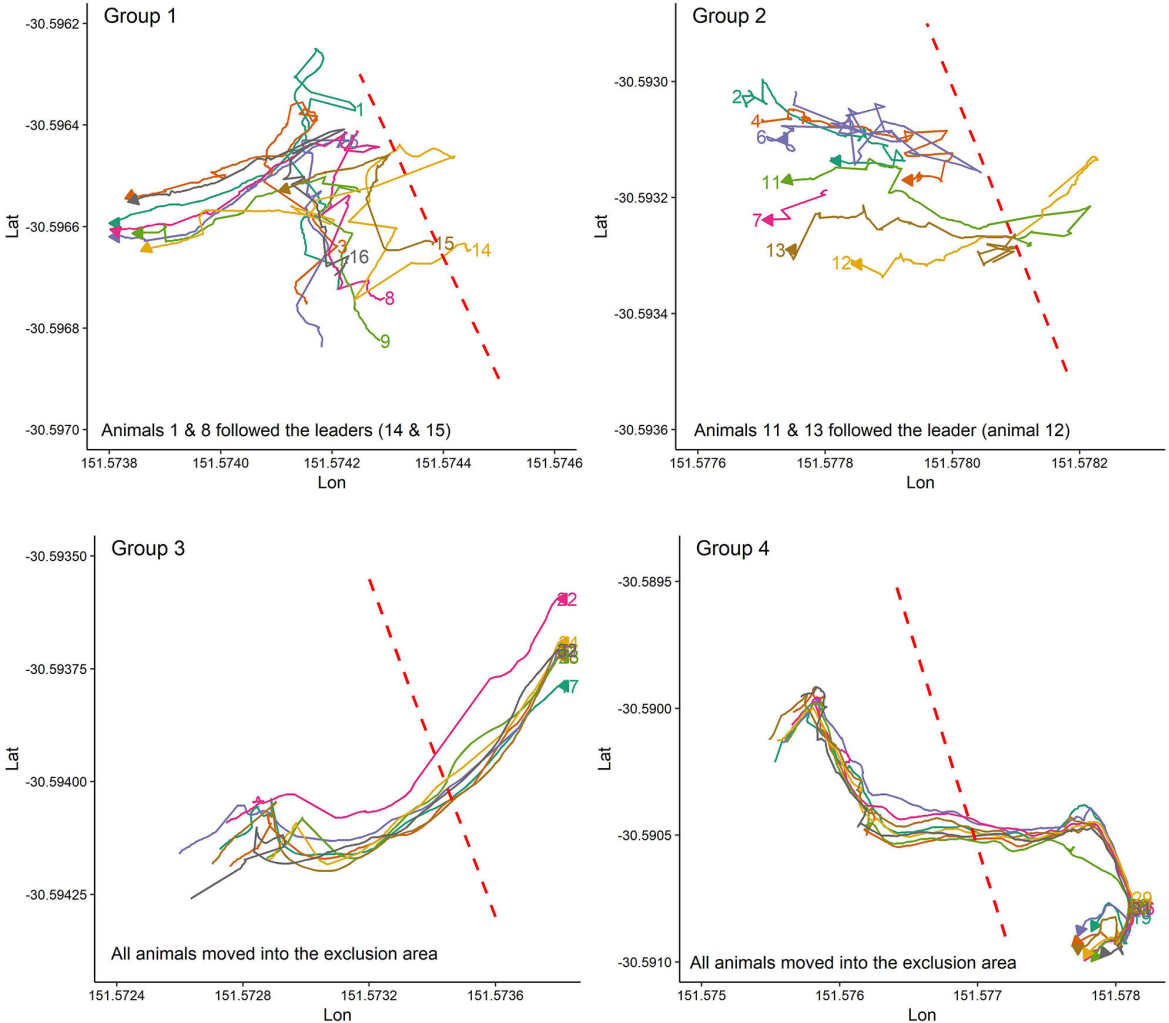
The animal's reactions during the first contact with the VF when animals have no experience with the VF. Plots are drawn across a period of 5 min.

Figure 3 illustrates some randomly selected examples of social facilitation during VF interaction events for animals in the first trial when at least some animals had experience with the VF. For instance, when animal 10 in group 2 or animal 5 in group 1 moved back into the inclusion zone following their received cues, other animals, even those with no experience with the VF (e.g., animals 9, and 16 in group 1; Figure 3) also moved back into the inclusion zone. In contrast, for the first interaction with the VF when no one had experience with the VF, almost all animals or those close (animals 11 and 13 in group 2) to the leader animal (s) followed him and moved into the exclusion zone (Figure 4).

**Figure 5:**
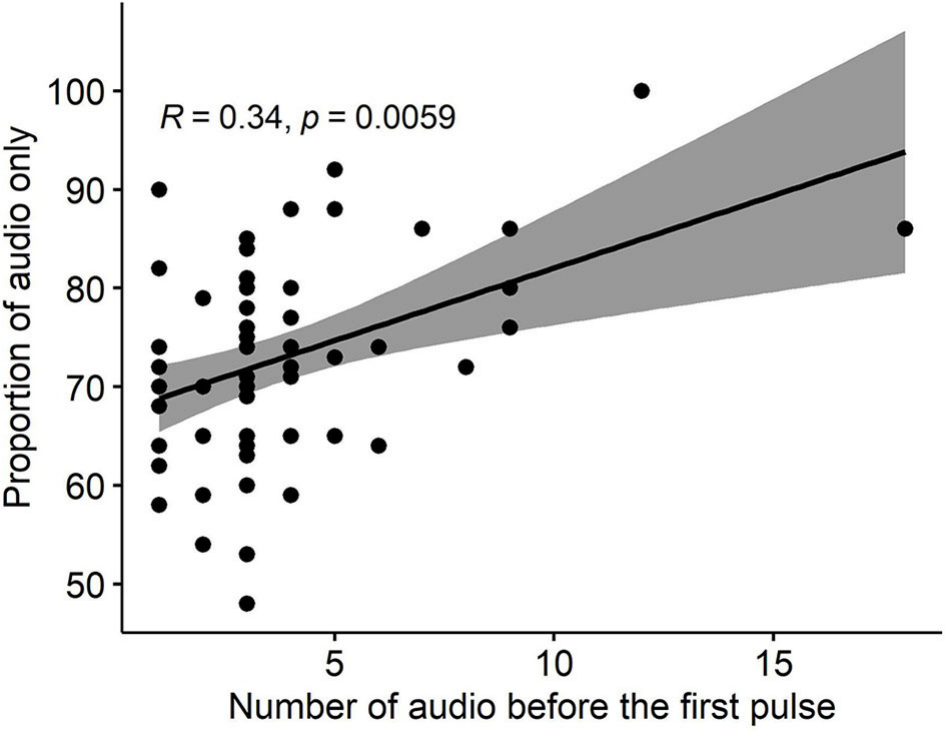
The correlation between the number of received audio cues before the first pulse and the proportion of “audio-only” cues (i.e., the proportion of all received audio cues that were not followed by a pulse) across 3 study days for all groups.

Almost every animal in each group (63/64) responded correctly to the audio cues (i.e., stopped or turned away thus avoiding an electrical pulse) before ever receiving their first electrical pulse, but to different degrees ranging from 1 to 18 audio cues before the first pulse (Table 4). There was a positive significant correlation (*R* = 0.34, *P* = 0.006) between the number of audio cues received before the first pulse, and the proportion of “audio-only” cues across the 3 days (Figure 5).

**Table 4:** Number of received audio cues before the first electrical pulse for individuals (1–8) in the studied groups.

	Received audio cue/Animals' number^*^								
**Trial/group**	**1**	**2**	**3**	**4**	**5**	**6**	**7**	**8**	**Mean/group**
**First trial**									
Group 1	18 (1)	3 (3)	1 (5)	3 (8)	2 (9)	3 (14)	6 (15)	3 (16)	4.9
Group 2	3 (2)	9 (4)	3 (6)	12 (7)	3 (10)	4 (11)	3 (12)	1 (13)	4.8
Group 3	3 (17)	4 (18)	2 (21)	4 (22)	3 (23)	3 (24)	5 (27)	1 (32)	3.1
Group 4	3 (19)	1 (20)	4 (25)	3 (26)	5 (28)	1 (29)	2 (30)	1 (31)	2.5
**Second trial**									
Group 5	1 (6)	3 (13)	6 (17)	1 (22)	1 (29)	3 (30)	8 (32)	4 (37)	3.4
Group 6	4 (2)	5 (14)	4 (19)	2 (20)	4 (35)	3 (36)	3 (38)	3 (39)	3.5
Group 7	3 (3)	3 (10)	1 (12)	3 (16)	7 (18)	3 (26)	9 (27)	–	4.1
Group 8	3 (4)	3 (5)	1 (7)	4 (9)	2 (23)	9 (24)	5 (25)	4 (34)	3.9

* *The numbers in parentheses are the ID of animals in each group*.

## Discussion

This study aimed to determine whether naïve beef cattle in small groups were socially facilitated in avoiding the exclusion zone in a virtual fencing (VF) system as well as in their associative learning between the audio and electrical cues. There were some individuals that first interacted with the VF more frequently, but there were no single animals that were consistently first within each group. Cattle showed clear patterns of social facilitation of movement behavior during VF interaction events where they stayed in the inclusion zone without receiving any cues themselves and turned away at the audio tone before ever experiencing the paired electrical pulse. This social facilitation of movement with a VF implemented was higher than movement facilitation during natural grazing patterns. This new evidence demonstrates that cattle can be influenced by each other during the implementation period of a novel agricultural technology.

The majority of interaction events with the VF occurred as a group with very few occasions where only one animal was by the fence line alone. This herding behavior is typical of cattle (6, 38) and enabled a group-level response to the VF where all animals stayed within the inclusion zone based on a few individuals receiving cues across each interaction event. That is, the studied cattle only had direct contact with the VF cues for 23.8% of the time, while for almost three quarters of the time they used the experience of other individuals to avoid the VF. However, there were no single individuals that always initiated the VF interactions. Some animals were more likely to be first to interact than others but with inconsistent patterns. Approximately 75% of events on average were led by three individuals within each group with more consistent patterns in some groups over others. These results are similar to previous observations of a single group of cattle exposed to a VF across a 10-day period where the frequency of being first to interact with the VF varied across individual cattle (30).

Some previous research has reported that dominant animals preferentially lead groups, such as when moving to new pasture areas (10). However, the relationship is typically non-linear, and no single individual shows exclusive herd leadership (9–11). This is consistent with the findings of the current study, but our assessment of leadership was restricted to one context. There was no assessment of the degree of influence these specific individuals may have had on group behavior in other situations (e.g., movement to a new grazing area, or movement to the water trough) and the relationship between VF leadership and dominance was not measured in these cattle. Thus, the animals were not identified as consistent leaders of the group but only as individuals of influence at the time of the interaction (39), limiting the conclusions regarding the influence that specific individuals may have on the behavior of other group members. Classification of dominance and quantification of the social dynamics within the group may provide further insight into patterns of facilitation where individuals differing in status, age, or social position may exert more influence on the group [e.g., in cattle (11); in chickens (40)]. Differences in some of these parameters could also account for the variation that was seen between groups in both the consistency of first interactors and degree of social facilitation (Tables 2, 3). Alternatively, personality differences may have affected interactions with the VF where bolder animals with higher motivation to explore and/or access the area in the exclusion zone may have initiated more VF interactions (41, 42). Regardless of the reasons for these individual differences, for VF technology to be successfully implemented, all cattle should wear neckband devices as no single individual of influence was identified.

The findings from this study show that adapting to a new technology can be facilitated by conspecifics when exposed to the VF system as a group. Previous studies of individual associative learning patterns (between the audio cue and electrical stimulus) during first exposure to the VF cues and avoidance learning (remaining within the inclusion zone) demonstrated high variation in both the rate of learning and the behavioral responses to the cues with some individuals running forward following an electrical pulse, and other animals turning away (28). When exposed as a group, the behavioral responses to the VF are more cohesive (i.e., all members of a group act in a similar manner), although they still vary between separate groups. This variation was particularly apparent in the very first experience with the VF where some groups of animals all received signals and broke through into the exclusion zone, compared with other groups that all turned around based on the experiences of only a few animals.

Facilitated or synchronized responses are typical of cattle (20–22) and were also shown in the current study during the control observations of natural grazing patterns. However, overall, comparatively more facilitation of movement was observed when the VF was implemented. Group-level responses in a potentially threatening situation are one of the benefits of group-living (43). Other research has shown cattle will act as a coordinated group in their patterns of avoiding biting pests (44, 45). In the case of the VF, the stimuli are initially highly unexpected with no visual cues and a benign audio tone as a warning for the electrical pulse. Avoidance based on the avoidance reactions of others can initially minimize an individual's experience of what may be observed as a negative experience of conspecifics when they suddenly react to the electrical pulse. The degree of reactivity by individuals receiving a pulse vs. unknown stimuli that instigated a change in direction during grazing likely resulted in the comparatively heightened facilitation of group members behavior in the presence of a VF (46). Subsequently, avoidance based on herd member's reactions can also minimize the frequency of moving into the exclusion zone and receiving electrical pulses. However, the VF eShepherd® system has been designed to be controllable and predictable for all individuals if they learn the association between the audio cue and electrical pulse (31). Through associative learning, all individuals can avoid receiving electrical pulses if they appropriately stop or turn away at the audio tone. In this study we demonstrated that individuals responded correctly to the audio cue multiple times without ever receiving an electrical pulse, indicating they were avoiding a benign stimulus, based on observations of conspecifics. The VF devices are designed to emit audio tones at a decibel level audible only to the animal wearing the device, although in calm conditions and close proximity, audio tones could potentially be heard by neighboring animals. It is thus likely that the cattle were associating their own audio tone with an avoidance response from watching the reactions of herd mates before they had received their own electrical stimulus for the first time. A similar observation has previously been stated for cattle learning to respond to a standard electric fence where animals avoided the fence without experiencing it themselves (47). In the current study, this facilitated response to the audio cues then resulted in some improvements in the rate of associative learning across the 3 day study duration but further research should elucidate the exact mechanisms behind this and whether the different types of learning (from watching others or self-experience) have any corresponding physiological and emotional impacts such as increased heart rate when learning is successful (48). The distinction between social facilitation and social learning and cognitive processes behind the observed behavioral patterns was unclear from the current study and future work should aim to determine the degree to which cattle may learn a VF system from observing others which could be achieved by exposure as a group followed by individual testing. Additionally, the number of assessed groups and group sizes were limited by available animals, paddocks, and pasture. Further testing across more groups and larger group sizes would confirm the degree to which successful implementation of a VF is influenced by social processes.

In conclusion, appropriately responding to virtual fencing technology is socially facilitated via observations of the reactions and behavior of other group members in beef cattle. In large commercial cattle groups, this could improve the effectiveness of the fence and minimize the number of electrical pulses each animal receives. Different animal groups vary in their behavioral reactions and learning rates, further assessment of group dominance hierarchies or social interactions may help understand the causes of these differences.

## Data Availability Statement

The raw data supporting the conclusions of this article may be made available by the authors to any qualified researcher, but only with the approval of Agersens Pvt. Ltd. to ensure commercial confidentially is maintained.

## Ethics Statement

This animal study was reviewed and approved by CSIRO FD McMaster Laboratory Chiswick Animal Ethics Committee (ARA18/25).

## Author Contributions

CL, DC, HK, and JL contributed conception and design of the study, and organized the database. HK performed the statistical analyses and wrote the first draft of the manuscript. DC, HK, and CL wrote sections of the manuscript. All authors approved the final version.

## Conflict of Interest

Agersens Pty Ltd. provided technical support with the neckbands but did not contribute to the study design, data analysis, or the decision to publish the results. The authors declare that the research was conducted in the absence of any commercial or financial relationships that could be construed as a potential conflict of interest.
